# Characterization and Environmental Evaluation of Recycled Aggregates from Construction and Demolition Waste in Belgrade City Area (Serbia)

**DOI:** 10.3390/ma17040820

**Published:** 2024-02-08

**Authors:** Filip Abramović, Miroslav P. Popović, Vladimir Simić, Vesna Matović, Radmila Šerović

**Affiliations:** 1 Environment and Sustainable Development Program, Singidunum University, Danijelova 32, 11000 Belgrade, Serbia; 2 Ministry of Environmental Protection, Government of Serbia, Omladinskih Brigada 1, 11000 Belgrade, Serbia; 3 Faculty of Mining and Geology, University of Belgrade, Đušina 7, 11000 Belgrade, Serbia

**Keywords:** construction and demolition waste (CDW), recycled aggregates, natural aggregates, environmental impact, substitute, landfill

## Abstract

Sustainable consumption of construction materials is an important segment of sustainable development goals towards reducing climate change. Since the consumption of natural aggregates raises environmental concerns, there is an increasing demand for use of recycled aggregates (RAs), as it enhances social and environmental benefits and creates a market opportunity. This paper presents the practice of using recycled construction and demolition waste (CDW) in the Belgrade city area (Serbia) as a resource. Two groups of CDW from Vinča landfill site near Belgrade are analyzed: raw material before, and RAs after, construction of a recycling facility on site. Comprehensive characterization is performed (including particle size distribution, density, water and organic pollutants content, various mechanical resistances, flakiness index, etc.) and compilation of samples analyzed and compared to show a holistic overview. The test outputs in both groups show acceptable values and meet required standards, indicating that recycled CDW generated in the Belgrade area can be used as a substitute to natural aggregates. In addition to that, the environmental and economic benefits from this use as a substitute are analyzed and discussed, proving the substantial income from sold Ras and the landscaping benefits, as well as ecological and economic benefits from energy savings.

## 1. Introduction

Stone aggregates are the most important building material in each country, being the basis of the infrastructure development of the society. In addition to road building [1], aggregates are used in the construction of housing and commercial buildings, as well as in industrial developments and various public infrastructure projects [2], which makes the aggregates sector the most important among the non-energy extractive industries [3,4]. Managing sustainable supply of aggregates at all levels is a very important task because of their great economic importance (in the EU, the contribution of aggregates in the mining sector was ~67%vol. and around 23% by value in 2013) [4,5].

Processing industries and consumers/users are limited naturally to the areas where there are deposits of aggregates, being also limited by existing quantities of natural aggregates and having always to take into consideration the economic criteria as well [6]. The area of interest in this study—the City of Belgrade (Serbia)—has enough aggregates for its needs at the moment, but neither management nor supply are coordinated within or across the area, and current use is not balanced with sustainable management of aggregates.

A sustainable use of aggregates is on the constant rise and has a much broader meaning [7,8,9,10], which makes it necessary to promote significant changes in patterns of consumption [11]. Therefore, there is a constant demand to search for alternatives, i.e., substitutes of natural aggregates, particularly among waste material [12,13]. It was assessed recently that approximately one-third of all waste generated in the EU comes from construction and demolition waste (CDW) [14]. Recycled CDW can be reused in various applications and materials, such as construction of roads [15,16], mixture for concrete or mortar production [17,18], secondary raw materials for brick [19,20], etc. This results in reducing carbon dioxide emission and waste production [15,21]. Although there have arisen disputes on whether the recycling chain causes higher energy consumption than landfilling [22,23], it turns out that processing CDW into added-value products (recycled aggregates) leads to environmental savings mainly through landfill avoidance, landscaping benefits, and nonrenewable resources preservation [16]. Various properties of CDW have been analyzed [12,13,14,17,20], partly comparing to natural aggregates [15,16], which has led to its detailed characterization for many aspects of its use, and classification of recycled aggregates into several types depending on CDW origins [24].

CDW is classified in the European Waste Catalogue 2000/532/EC [25] and, according to the Waste Framework Directive 2018/851 [26], recycling percentage of this waste must be 70% by 2020. In 2020, the total waste generated in the EU by all economic activities and households amounted to 2135 million t or 4815 kg per capita [27], and CDW in EU 27 averaged 37.5% of total waste generated, while in Serbia it was 1.2%. However, the recovery rate of CDW in the European Union (EU-27) was 88 percent in 2018 [28]. Provisional estimates of aggregates production data for 2019 revealed that total recycled aggregate production in Europe (42 countries) was 288 Mt [29], or 6.9% of total aggregate production. The largest producers of recycled aggregates in Europe are UK, France, and Germany, with 12–24% of total aggregate production.

Following the EU regulations, this Directive [26] was adopted and incorporated into the legislation of Serbia [30]. Subsequently, the city of Belgrade has adopted a local waste management plan for the city of Belgrade 2021–2030, which has determined the location for a recycling plant and estimated annual CDW quantity there to be nearly 500,000 t [31]. In 2020, around 729,000 t of CDW were generated [32]. Most of the waste from construction and demolition is disposed of or, more often, disposed of together with municipal waste in municipal landfills. The estimated composition of construction and demolition waste is as follows: soil excavation, 75%; construction and demolition waste (ceramics, concrete, iron, steel, plastic waste), 15–25%; waste asphalt and concrete, 5–10% [33]. The collection and disposal of construction and demolition waste is subject to the polluter pays principle, which means that the waste producer is solely responsible for the legal and safe disposal (final disposal or recycling) of the waste generated. The existing infrastructure for the treatment of construction and demolition waste in the Republic of Serbia includes only a few crushing plants and recycling of less than 1000 t of waste per year. In Belgrade, a construction and demolition waste treatment plant was built in Vinča, with a capacity of up to 200,000 t per year, and is in trial operation [33].

The represented and expected amounts, having also a positive trend in increase, undoubtedly indicate the great potential of the use of CDW as a substitute of natural stone aggregate and decrease the need for its exploitation. This may drastically diminish the environmental degradation caused by quarry exploiting (as the main actual practice), also protecting the biotopes from devastation and species from extermination, and preserving stone aggregates as a resource for future. In addition to this component (nature protection and sustainable development), the use of these substitutes can prevent the environment from undergoing pollution by depositing the waste onto illegal or registered landfill sites and from further soil and water contamination by leaching dangerous and other components from it. Attention should be paid also to the materials less present as components of CDW, such as metals, wood, and plastics. Revitalization of metals is needed mainly for their market value. After sorting, wood can be chopped into woodchips for chipboard production, producing the income that can be calculated from the mean actual market price [34].

The economic importance and potential socioenvironmental impact of their production and manufacturing are crucial for the city of Belgrade’s growth, wherefore they must be produced and used there according to sustainable development principles. In the city of Belgrade area, CDW originates from maintenance, construction activities, or demolition of buildings and civil infrastructure works, as well as from construction and demolition of roads. It includes concrete, cement, and various mortars, conglomerates and mixed bituminous bricks, tiles and blocks, excavation soil, wood, paper, cellulose, polystyrene, metals, plastics, chalk, ceramic, glass, asbestos, etc. By now, there are scarce studies focusing on the CDW and recycled aggregates (RAs) processing and practice in Serbia; moreover, focusing on its districts/cities [4,5,22].

In this study, CDW from Vinča landfill site in Grocka municipality area (City of Belgrade, Serbia) was analyzed in order to investigate the possibility for it to be used as a substitution for natural aggregates, and it was subsequently compared to recycled CDW from the newly installed recycling facility. One goal was to compare the physical and mechanical properties of RAs with natural aggregates from Serbia, as well as categorization of RAs according to EN 12620 [35] and EN 13242 [36]. The other goal was to perform an assessment of a range of potential environmental and socioeconomic benefits emerging from this use as a substitution.

## 2. Materials and Methods

### 2.1. Methods of Sampling and of Sample Preparation from Landfill Site

**Figure 1:**
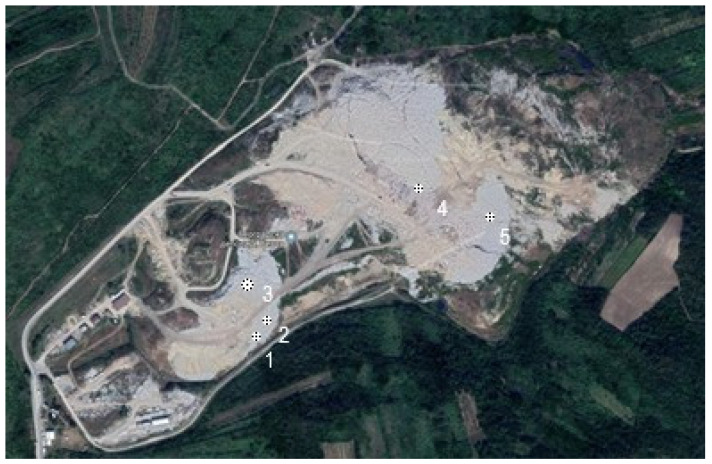
Five locations of CDW sampling in Vinča repository, Belgrade (Serbia).

Sample acquisition was carried out from CDW on five different locations (represented in Figure 1) of Vinča landfill site in Grocka municipality area, and five samples of 2500 kg—giving a total mass of 12,500 kg—taken for analysis in 2018.

**Figure 2:**
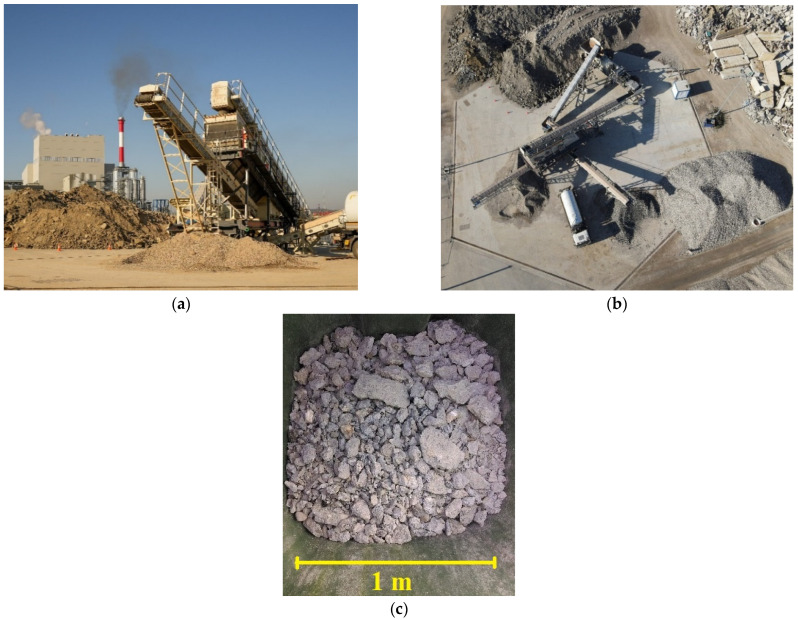
(**a**) CDW crusher in Vinča; (**b**) aerial view of facility; (**c**) crushed material prepared for analysis (photos from 2023 [37]).

An Arjes Impaktor 250 shredder machine (commonly used for concrete milling) produced by “Arjes”, Leimbach, Hessen (Germany) was utilized in this study for CDW crushing, whose crushing capacity ranges from 80 to 100 t of reinforced concrete per hour. A mass of 882 kg of steel was separated from bulk during crushing, while 1120 kg of wood and 600 kg of ceramics were removed from bulk before placing it into the machine. The crushing process is shown in Figure 2a,b.

Five separated samples of crushed material (an example is shown in Figure 2c), each one weighing 200 kg, were secondarily crushed and analyzed in the Laboratory of Stone and Stone Aggregates of the Highway Institute, Belgrade, Serbia. Secondary crushing was carried out in an impact crusher, type UG-2 (manufactured in 1960s by the Highway Institute, Belgrade, Serbia, for internal use). The maximum size of pieces of recycled concrete samples before the start of secondary crushing was about 30 cm. The granulometry of the samples corresponded to the fractions 0/125 and 0/63 mm after crushing.

### 2.2. Methods of Analyzing Samples from Landfill Site

The analyses of crushed samples by (1) “Los Angeles“ abrasion test machine was performed (according to the European standard EN 1097-2 [38], labeled as “LA”), followed by (2) determination of the loose bulk density of dry aggregate, *ρ_b_* (according to European standard EN 1097-3 [39]), (3) granulometric analysis (European standard EN 933-1 [40]), (4) determination of aggregates contamination/pollution by organic matter (labeled as OM; European standard EN 1744-1 [41]), (5) determination of wear resistance (*M*_DE(10–14)_, standard EN 1097-1 [42]), (6) determination of apparent density of particles (*ρ*_a_), density of dried grain (*ρ*_rd_), actual saturated-surface-dry density of grain (*ρ*_ssd_), and water absorption in aggregates, WA_24_ (based on standard EN 1097-6 [43]), (7) determination of particle shape—flakiness index (*FI*, standard EN 933-3 [44]), (8) crushing resistance determination by Treton impact test (*T*, national standard SRPS B.B8.019/1961 [45]), (9) frost-resistance test (*FR*, national standard SRPS B.B8.044:1982 [46]) and (10) determination of crushability by cylinder compression test method (*CCTM*, standard SRPS B.B8.033:1994 [47]).

### 2.3. Methods of Preparation and Analyzing Samples from the New Recycling Facility

During the material transport, the document of waste movement tracking is used as a proof of material origin and its composition in order to be able to confirm the sampling location. Inside the new recycling facility constructed at the former landfill site, additional analyses of samples are regularly performed according to regulatory rules of Republic of Serbia and legislative acts and recommendations of European Union. In 2022, two samples were taken directly from the trucks coming from the demolition site, i.e., the first author was supervising the whole process of taking samples. Samples were analyzed in the Centre for Roads and Geotechnics of the IMS Institute, Belgrade. The analyses included identification and classification along with physical and mechanical tests, as follows: (1) determining the water content in materials (European standard EN ISO 17892-1 [48]), (2) determining the particle size distribution (European standard EN ISO 17892-4 [49]), (3) determining the content of combustible and organic matter (Serbian standard SRPS U.B1.024:1968 [50]), (4) determining the dry density to water content ratio—modified Proctor test (European standard EN 13286-2 [51]), and (5) determination of California bearing ratio (European standard EN 13286-47 [52]).

## 3. Results

Compilation and analysis of the two sets of samples were performed, as explained previously, and compared to technical regulations and standards.

**Figure 3:**
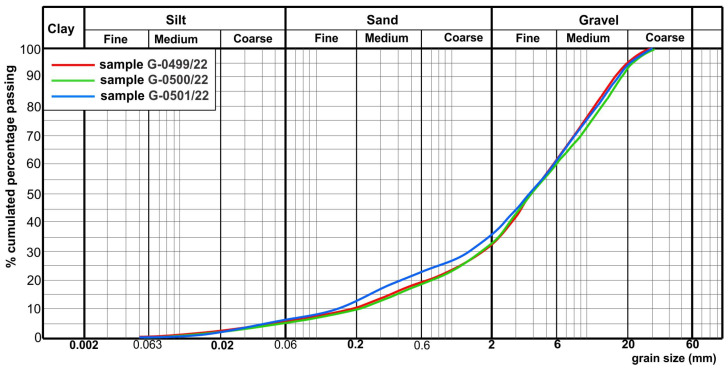
Grain size distributions in three analyzed samples shown in Table 3. Legend: sample #1—sample code G-499/22; sample #2—sample code G-500/22; sample #3—sample code G-501/22.

Results of the grain size distributions of crushed samples are given in Table 1 and visualized in Figure 3, while those of the tests (1), (2), and (4)–(10) from Section 2.2 are listed in Table 2. Results of the analyses of recycled CDW samples from the new Vinča recycling facility are represented in Table 3 and Table 4.

**Figure 4:**
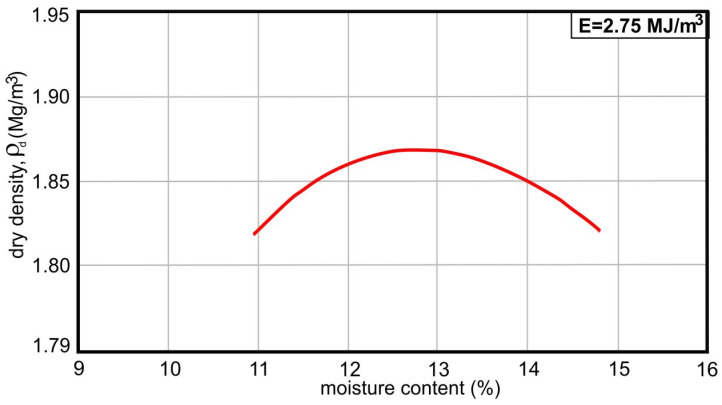
Proctor test results in recycled aggregate: dry density as a function of moisture content, in sample #3 (sample code G-0501/22, see Table 4). Applied mechanical energy in the test (for soil densification by air removal in voids and soil particles rearrangement): 2.75 MJ/m^3^.

Particle size distribution of all analyzed samples indicated a high participation of the coarse aggregate fraction. In samples 1–5, the content of particles with size above 2 mm varied within 80–95%, while the participation of the same particle size in samples G-0499-0501/22 was more uniform (64–68%). These ones were characterized with the higher amount of fine particles (<0.063 mm), being no more than 3% in samples 1–5. Results of density evaluation showed that oven-dried particle density for all crushed samples (1–5) was uniform (2.33–2.34 Mg/m^3^) in all fractions. As represented in Figure 4, maximum dry density of 1.87 Mg/m^3^ was reached at optimal moisture of 12.8% in sample 3 from the new recycling facility in Vinča (sample code G-0501/22). Water absorption was detected higher in fractions of 4/8 mm, as expected, where it was 4.87%, while in coarse fractions it varied from 4.28 to 3.81%.

The mean value of percentage mass loss (10.9%) after frost resistance test showed low resistance to the effects of frost. The results of shape analysis with mean value of 13 determined the low content of grains with an unfavorable shape. The resistance to wear of samples determined by the micro-Deval coefficient and LA abrasion resistance with maximum values of, respectively, 25 and 34 indicated good resistance to wear and fragmentation. Crushability index (CCTM) determined by cylinder compression (under 40 t) test method was the smallest in the 4/8 mm fraction and expected to be the highest in the 16/31.5 mm.

The optimum moisture content of sample 3 (sample code G-0501/22) was obtained as 12.8% and the maximum dry density was determined as 1.87 M/cm^3^ according to the laboratory modified Proctor test. The content of organic impurities in aggregate may not color the 3% NaOH solution darker than the referent color, which was satisfied in analyzed samples (Table 4).

## 4. Discussion

### 4.1. Characterization and Possible Utilization

The physical and mechanical properties of recycled aggregate are mandatorily used to characterize the quality of RAs and for defining the possibility of their proper use. The main standards used in results and requirements comparison were EN 12620 [35] and EN 13242 [36] and national technical regulations.

According to the grading requirements of standard EN 12620 [35], samples 1–5 are all-in aggregates, but they could not be designated by G_A_ category due to D > 45 mm. According to the requirements of the standard EN 13242 [36], samples 1–5 are coarse aggregates, designated by G_C_80-20. The content of particles finer than 0.063 mm is limited to the application of recycled aggregates as the road construction materials. It makes this property an extremely important parameter for each aggregate purpose. The amount of fine particles ( ≤ 0.063 mm diameter) may participate in the aggregate for unbound bearing layers up to 5% in landfill, i.e., up to 8% after incorporation, which was realized in analyzed samples (Figure 3, Table 3). Single results of analyzed samples taken from the landfill have values up to 3% (except sample 3), so the fines content can be considered as nonharmful. Samples can be declared as *f*_3_, while sample 3 is classified in category *f*_11_ (according to EN 12620 [35]), respectively, in category *f*_5_ (according to EN 13242 [36]). Percentage of grains of up to 0.02 mm diameter size in the aggregate may not exceed 3% according to the national technical regulations, and all analyzed samples met this requirement.

The flakiness index is an aggregate property that largely depends on the type and method of crushing. It affects the interlocking of the aggregate particles and slightly affects properties of later-produced hardened concrete [55]. According to this property, all tested samples have relatively low values that determine them into the category *FI*_15_, excepted sample 1, which belongs to category *FI*_20_. The percentage of grains that do not meet l:d ≤ 3:1 requirement may be 20% at most to national technical regulations (category *FI*_20_ according to EN 13242 [36]).

It is known that recycled aggregates have a lower particle density compared to the natural aggregates which varies between 2.3 and 2.9 Mg/m^3^ [56]. Based on the obtained particle density values, the examined fractions of the recycled aggregate have a specified property very close to the density of the natural aggregate. The declared value of oven-dried particle density is 2.34 Mg/m^3^ for studied fraction recycled aggregates (sample 1–5). It satisfies the minimum density value of oven-dried particles 2000 kg/m^3^ for production of all concretes (EN 12620 [35]) in conformity with EN 206-1 [57].

**Figure 5:**
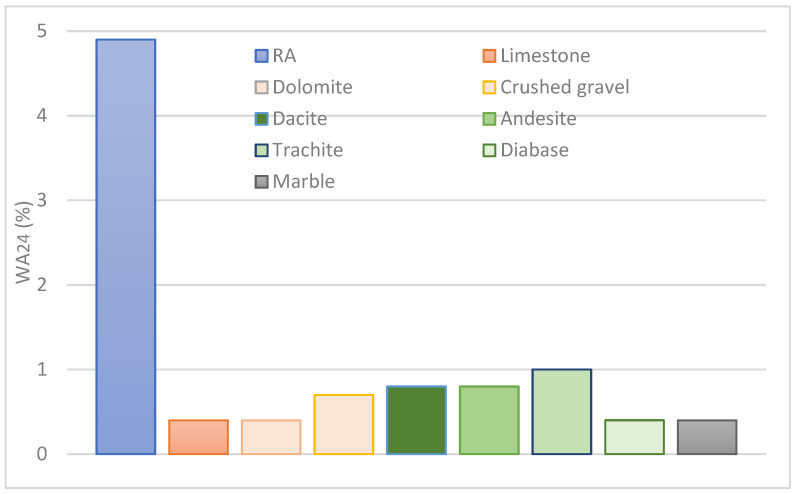
Histogram of water absorption (WA_24_) mean values of recycled aggregate (fraction 4/8 mm of sample 1–5) compared with mean value for natural aggregates [58].

Water absorption (WA_24_) is considered an important physical property of recycled aggregates [55]. It characterizes the voids’ volume in the aggregate available for water and it is an indirect precursor of frost resistance. Water absorption of 4.87% in fraction 4/8 mm of the investigated recycled aggregates is expected to be higher than that of the natural aggregates produced from magmatic, sedimentary, and metamorphic rocks in Serbia (Figure 5). Data in the literature demonstrate that average WA_24_ values of numerous analyses of natural aggregates are up to 1%, while the maximum obtained values rarely exceed 2% [58]. Generally, WA_24_ for natural aggregates varies from 0.5% to 1.5% [56].

It is also known that the water absorption of recycled aggregates largely depends on binder content, cement paste content, and presence of mortars [59,60]. Our result of WA_24_ for RAs are significantly and expectedly higher than the WA_24_ of natural aggregates, but they are in agreement with results from the literature for the coarse fraction of 300 RCA samples, which ranged from 1.0% to 15.0% with an average of 4.9% [56], and results of experimental investigation carried out on RCA samples with WA_24_ of 4.6% [55].

Taking into account the results of water absorption and mass loss of 9–15% after five cycles with sodium sulphate solution (test method by Na_2_SO_4_ solution; SRPS B.B8.044:1982 [46]), the analyzed samples cannot be freeze–thaw-resistance or sulphate-soundness categorized according to EN 12620 [35].

**Figure 6:**
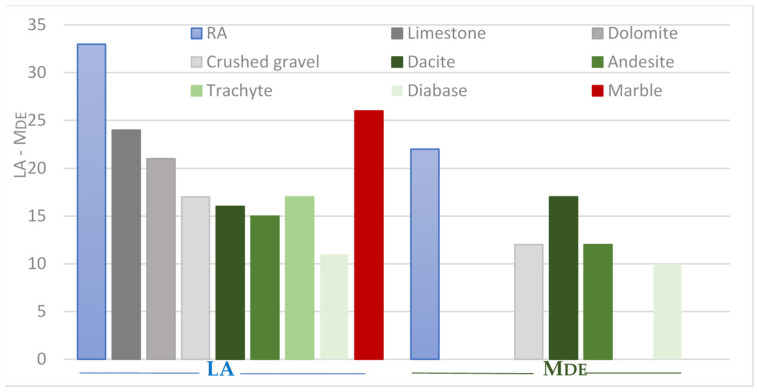
Histogram of abrasion resistance (LA) and micro-Deval coefficient (M_DE_) mean values of recycled aggregate (sample 1–5) compared with mean values for natural aggregates [58].

Mechanical properties of studied recycled aggregates samples are expressed through Los Angeles and micro-Deval coefficients, and crushability index CCTM too. The measured values of the LA index are, on average 33, and, respectively, 22 for the micro-Deval coefficient. In Serbia, natural aggregates produced from magmatic or sedimentary rocks and used for concrete or road construction exhibit LA values of less than 25% (see Figure 6).

**Figure 7:**
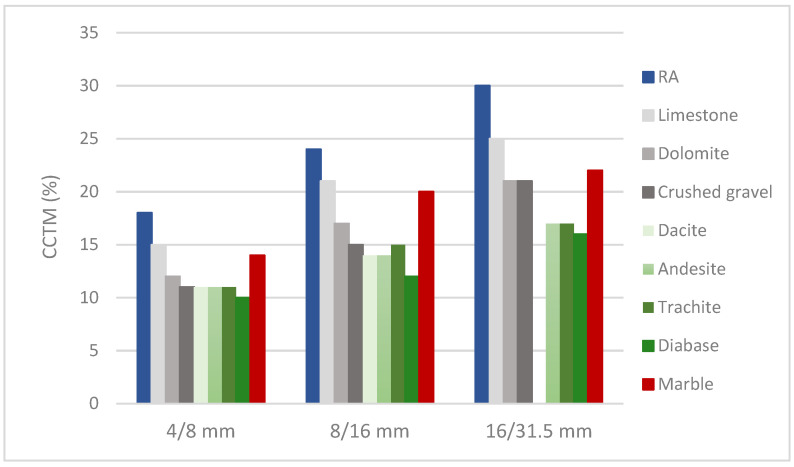
Histogram crushability index (CCTM) mean values of recycled aggregate (sample 1–5) compared with CCTM mean values for natural aggregates [58].

Although the LA index values of the tested RAs are higher than natural aggregates, they indicate good resistance to fragmentation. The obtained values LA are also in agreement with studies conducted on 111 RCA samples (crushed materials from construction and demolition wastes), with an average LA of 33%, or that of 51 MRAs (crushed construction and demolition wastes) samples, with an average of 36% [56]. Unlike the LA index, the results of studies on the MD index of recycled aggregates are few in the scientific literature, but they all agree that the wear index is higher than the same for natural aggregates [55], as is the case in our study. The obtained mean value of the M_DE_ index is coherent with the values for RCA of 24% [55]. Crushability index (CCTM), as an important aggregate property for concrete use, is higher in all fractions than in natural aggregates (Figure 7), but still low enough and, thus, sufficient for the national quality criteria for concrete aggregates.

According to resistance to wear and fragmentation coefficient values, all samples are declared as *LA*_35_ category until *M_DE_* index indicates the category *M_DE_*25. The percentage of crushed stone grains in the mixture for unbound bearing layers has to be determined as a part of the procedure of dimensioning road construction by using the distributions from the standard. According to national technical regulations, crushing resistance of aggregate, determined by LA method for unbound bearing layers in roads, may be at most 30% for medium and high traffic load, i.e., at most 35% for low traffic load. The tested samples satisfy this last condition. 

The content of organic impurities in aggregate may not color the 3% NaOH solution darker than the referent color, which was satisfied in analyzed samples. Aggregates for unbound bearing layers may not contain inappropriate grains.

California bearing ratio (CBR) must be (1) at least 40% (or more) for natural and mixed aggregates in which less than half of the grains are crushed ones, and (2) at least 80% (or more) for crushed and mixed aggregates in which less than half of the grains are crushed ones. This requirement was met as well (see Table 4).

Mixtures of coarse/crushed stone grains (rarely a mixture of these with crushed secondary resources) are commonly used as basic materials in unbound wearing-out layers. Fractions of 0/22 mm, 0/32 mm, and 0/45 mm are used as basic ones, and fractions of 0/8 mm for NHS surface. Granulometric composition of the basic fractions of stone grains mixture has to secure the best basement possible. There should be a majority of larger-sized stone grains in the mixture.

### 4.2. Selling and Landscaping Benefits

The main part of CDW originates from minerals and can be used (in the form of recycled aggregate) primarily in road construction [61]. This also provides the reduction in primary construction materials use. The goal should be to reach a closed-loop cycle in which a recycled aggregate would have the same use as a primary aggregate, i.e., as the main component in concrete fabrication (with the possibility of additional refinement). In the other applications, recycled aggregate acts as a component of the other construction materials or mixtures. These achievements are a particularly important point in the process of CDW management.

It is assumed that only 50% of fractions could be sold, while the rest would be used for covers, leveling, and filling the devastated areas. The income achieved from selling received and collected CDW fractions after the refabrication in crushing facilities can be calculated from the Q4-2023 average price of EUR 3/t [62].

Landscaping benefits (as already pointed out, according to the action plan) refer to the soil generated in excavations on the construction sites in Belgrade, and could be used for filling the devastated areas, i.e., for ecosystem restoration and landscape arrangement. Social benefits achieved in this way comprise regulation of air pollution–carbon sequestration, reducing damages from floods, temperature regulation, a possibility for recreational area arrangement, positive health effects etc. According to [7], the value of these benefits is assessed to be EUR ~3121/ha. Supposing that up to 1 m height (average) is being covered, it is possible to cover ~67 ha of devastated areas annually with the amount of ~1 million t.

### 4.3. Assessed Environmental and Economic Benefits from Energy Savings

There are various economic and environmental benefits from recycling processes. Studies in the EU show that the potential of a circular economy in the construction and environment sector particularly comes from (1) reducing feedstocks, energy, and water used in production of a certain good (product) instead of using recyclables, (2) reducing greenhouse gas (GHG) emission, (3) employment increase, (4) reduction of CDW amount deposited (i.e., reducing the empty area occupation in landfill), (5) reduction of landscape degradation, and (6) reduction of natural aggregate extraction and preserving nonrenewable resources. The production of 1 t of recycled aggregate requires 23% less energy than the aggregate production from natural resources [8].

Some important recommendations for CDW and production of recycled aggregates [63] may enhance the recycling process. The methodology applied for demolition may significantly affect the performance of the subsequent recycling process and the properties of the recycled products. Presorting of CDW at construction (demolition) sites may reduce recycling or disposal costs and ensure better quality for the recycled products. RAs are mainly used as bulk aggregate for road and railway foundations or for environmental restoration, and for preparation of the low-strength concrete mixtures. To secure the market of recycled aggregates, their price must be at least 20% lower compared to the price of natural aggregates, which is impossible in case of Belgrade, which has large amounts of total aggregate supply of natural sand and gravel from the Danube River [5].

Knowing that the production of 1 t of recycled aggregate demands 13.17 kWh, compared to aggregate production from natural resources, which demands 17.20 kWh, a difference of 4.03 kWh was taken into account, along with 1 kWh price in Serbia in 12/2023 of RSD 14.29 = EUR 0.12 (after tax) [64]:

4.03 kWh (from 1 t of recycled material) × EUR 0.12/kWh = EUR 0.4836(1)

Based on studies on carbon content in materials [65,66], it is assessed that every type of construction material can cause between 0.07 and 0.22 t of emitted carbon dioxide. In this slightly conservative approach, it is assumed that 0.15 t of CO_2_ is contained in every reduced type of construction waste (reused material). Environmental advantages were assessed by using economic prices of CO_2_ equivalent given by European Investment Bank for the 2020–2050 period, and the range of these prices in 2050 is from EUR 80/t to EUR 800/t. 

Recycling of CDW is also important from the point of lifecycle of aggregates, as it ensures transition from inert waste to final product in the following way: inert waste–recycling–recycled aggregates–construction products [63]. Closing the loop from production to waste and increasing material reuse and recycling would decrease the demand for feedstocks and assist in alleviating both prices’ instability on the feedstock market and supply risk. Feedstock expenditure is often used as a replacement for the ecological pressures of resources use. It measures the resources directly spent inside a national economy, understanding that every type of material that enters the economy would eventually exit either as waste or emission.

In this assessment, escaping the ecological damage (caused by resources extraction) was assessed by the average price of deposition in Serbia, being EUR 15/t for every extracted tone of replaced feedstock [31].

## 5. Conclusions

By analyzing the results obtained from the examination of both groups of samples and comparing the characteristics with imposed standards for materials used in construction, as well as by the assessment of environmental and economic benefits, the following conclusions can be made:

(1) Materials obtained by recycling the construction waste generated in the Belgrade area are in accordance with the aforementioned imposed propositions.
(2) These materials can be used as a substitute of natural aggregates: as unbound bearing layers, as unbound wearing-out layers, and as sealing/strengthening aggregates, as well as cement-containing concrete mixtures.
(3) There are significant selling and landscaping potential benefits from using the recycled CDW.
(4) A notable environmental and economic benefit can be achieved by nearly EUR 0.5/t savings (and 23% less energy consumption) from recycled aggregate use instead of aggregate production from natural resources.

## Figures and Tables

**Table 1:** Granulometric analysis of crushed samples.

Sieve (mm)	Sample No.				
	1	2	3	4	5
	Cumulative Passing (%)				
120	100	100	100	100	100
90	100	100	100	100	100
63	80	95	90	93	93
31.5	58	78	69	66	64
16	43	51	57	49	42
8	27	29	41	32	19
4	16	16	26	18	9
2	10	10	19	11	6
1	7	6	12	6	4
0.5	5	5	9	4	3
0.25	3	3	6	2	2
0.125	3	3	4	2	1
0.063	2.2	2.3	3.7	1.2	0.8

**Table 2:** Analysis of the specific parameters of crushed samples.

Property			Sample No.					Mean Value
			1	2	3	4	5	
*ρ_b_* (Mg/m^3^)			1.50	1.50	1.56	1.45	1.38	1.48
*ρ_a_*	(Mg/m^3^)	4/8	2.63	/	/	/	2.64	2.64
*ρ_rd_*			2.33	/	/	/	2.33	2.33
*ρ_ssd_*			2.45	/	/	/	2.44	2.45
*ρ_a_*		8/16	2.59	/	/	/	2.62	2.61
*ρ_rd_*			2.33	/	/	/	2.34	2.34
*ρ_ssd_*			2.43	/	/	/	2.45	2.44
*ρ_a_*		16/32	2.53	/	/	/	2.64	2.59
*ρ_rd_*			2.32	/	/	/	2.33	2.33
*ρ_ssd_*			2.40	/	/	/	2.44	2.42
WA_24_ (%)		4/8	4.81	4.80	4.81	4.81	5.12	4.87
		8/16	4.18	4.17	4.18	4.18	4.69	4.28
		16/32	3.51	3.50	3.51	3.51	5.05	3.81
FI (%)			16	13	11	14	9	13
OM (%)			/	/	/	/	/	/
FR (% loss of mass)			9.5	9.0	14.8	10.1	11.1	10.9
LA			34	31	31	34	34	33
M_DE_			23	23	25	21	19	22
T (%)			22.65	21.03	22.04	26.38	23.58	23.14
CCTM (%)		4/8	18.25	19.24	20.81	17.41	16.64	18.47
		8/16	26.32	24.54	25.65	23.12	21.86	24.29
		16/32	30.17	29.12	30.91	28.75	30.54	29.89

Legend: *ρ_b_*—the loose bulk density of dry aggregate; *ρ_a_*—apparent density of particles; *ρ*_rd_—density of oven-dried grain; *ρ*_ssd_—actual saturated-surface-dry density of grain; WA_24_—water absorption; FI—flakiness index; OM—organic matter; FR—frost resistance test; LA—Los Angeles index; M_DE_—wear resistance index; T—Treton impact test; CCTM crushability by cylinder compression test method.

**Table 3:** Analyses of CDW samples treated in the new recycling facility in Vinča (SRB).

Soil Samples Data			Fraction (%)				Examinations							Classification EN ISO 14688-2:2018 [53]	General Classification SRPS U.B1.001 [54]
CDW Sample No.	Sample Code	Sample Class (1–6)	Granulation/mm				Moisture, w (%)	% of Grains <0.063 mm	d_10_	d_30_	d_60_	$C_{u}=\frac{d_{60}}{d_{10}}$	$C_{c}=\frac{d_{30}^{2}}{d^{60}d^{10}}$		
			Clay <0.002	Silt 0.002–0.06	Sand 0.06–2.00	Gravel 2.00–64.0									
1	G-0499/22	3	1	5	26	68	5.8	6.0	0.19	1.75	5.60	29.47	2.88	GW-GM	sacIGrW
2	G-0500/22	3	1	4	28	67	5.2	5.1	0.21	1.71	5.99	28.52	2.32		
3	G-0501/22	3	1	6	29	64	5.8	6.8	0.13	1.31	5.51	42.38	2.40		

**Table 4:** Results of the tests performed on sample # 3 (sample code G-0501/22 in Table 3).

CDW Sample No.	Dry Volumetric Mass (t/m^3^)	Moisture Content during Preparation (%)	Burnable Matter (%)	Organic Matter (%)	Proctor Test			CBR (%)
					Dry Density, *ρ*_d_ (Mg/m^3^)	Dry Unit Weight, γ_d_ (kN/m^3^)	Optimal Moisture Content, w (%)	
3	1.861	12.3	7.6	1.7	1.87	18.33	12.8	111

## Data Availability

The data supporting reported results can be found by contacting Mr. Filip Abramović (first author) and at the Highway Institute, Belgrade (Bulevar Peka Dapcevica 45, Belgrade 11000, https://www.highway.rs/ (accessed on 4 January 2024)).
